# A review of novel research technology to explore the mystery of traditional Chinese medicine: Terahertz

**DOI:** 10.1097/MD.0000000000035870

**Published:** 2023-11-17

**Authors:** Shao-hui Geng, Li Liu, Zhi-min Lin, Hui Zhang, Ri-geng Mei, Xin Liu, Jian-cheng Liu, Guang-rui Huang, Wen-chun Zhang

**Affiliations:** a School of Life Sciences, Beijing University of Chinese Medicine, Beijing, China; b School of Acupuncture-Moxibustion and Tuina, Beijing University of Chinese Medicine, Beijing, China; c School of Chinese Materia Medica, Beijing University of Chinese Medicine, Beijing, China; d School of Traditional Chinese Medicine, Beijing University of Chinese Medicine, Beijing, China; e School of Traditional Chinese Medicine, Jiangxi University of Chinese Medicine, Nanchang, China.

**Keywords:** Meridian and acupoints, moxibustion, Qi, Terahertz, traditional Chinese medicine theory

## Abstract

During the 2022 Annual National Terahertz Biophysics Conference, the hypothesis was proposed that bio frequency electromagnetic fields sensitive points, akin to acupuncture points, exist in the human body. This development has prompted numerous researchers to apply terahertz technology to the field of traditional Chinese medicine (TCM). In recent years, terahertz technology has achieved notable progress in the field of TCM, particularly concerning the meridian-collateral system. This review systematically presents the advancements in terahertz technology and its implications on TCM theory from a biophysical perspective. Additionally, it summarizes the utilization of terahertz waves in elucidating aspects of TCM, particularly focusing on the scientific connotation of Qi, the theoretical foundation of the meridian-collateral system, and moxibustion in diagnosing and treating diseases. We aimed to explore the innovative applications and distinct advantages of terahertz technology in TCM and its feasibility as a pioneering technological tool for the modernization of TCM.

## 1. Introduction

Terahertz (THz) waves encompass frequency bands ranging from 0.1 to 10 THz with corresponding wavelengths spanning from 3 to 0.03 mm, signifying a transition from macroscopic to microscopic photonics.^[1]^ THz waves hold significant potential for application in biomedicine’s diagnostic and imaging domains due to their low photon energy and, thus, are being non-ionizing and noninvasive. During the 2022 annual Terahertz Biophysics Conference, academician Guozhi Liu proposed the hypothesis that “sensitive spots” within the human body could be influenced by electromagnetic fields within biological frequency bands, including acupuncture points.

**Figure 1.**
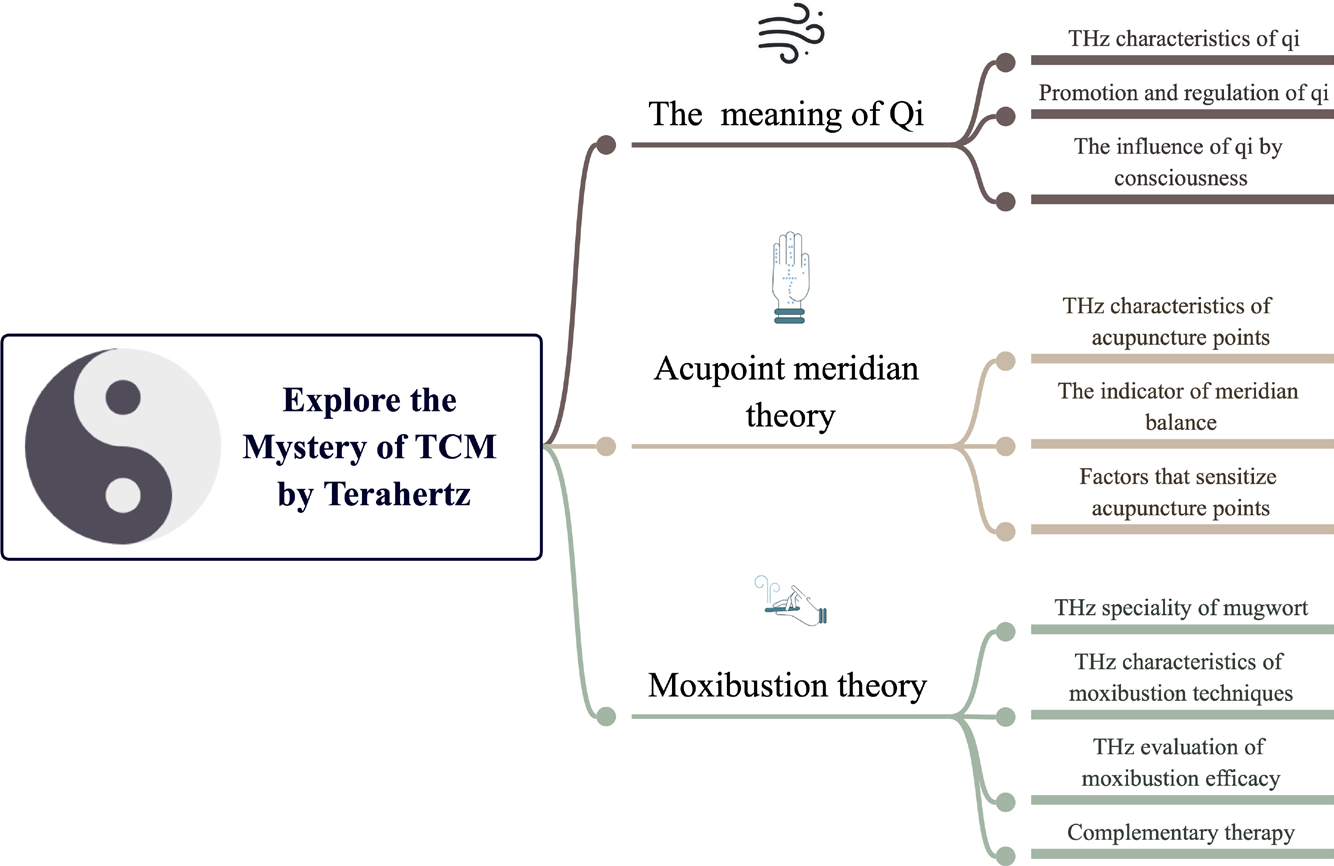
Application of THz technology in TCM theory research. TCM = traditional Chinese medicine, THz = Terahertz.

Significant advancements in the study of meridians in traditional Chinese medicine (TCM) have been facilitated by the application of THz technology. As an illustration, THz technology has been employed to elucidate contemporary mechanisms underlying TCM theories^[2]^ and to investigate disease treatment through the combined use of THz and acupuncture.^[3]^ In this review, we summarize the THz technology’s applications to unveil the scientific connotations of TCM theories. This encompasses 3 key aspects (Fig. 1): validation and substantiation of the presence and role of “Qi” in TCM theories; revelation and quantification of the thermo-effective relationship between moxibustion and disease treatment; and exploration of the material foundations and spectral information associated with meridians and acupoints.

## 2. Overview and characteristics of THz technology

THz waves are electromagnetic waves commonly occurring in nature, situated between microwaves and infrared light waves. THz waves exhibit distinctive characteristics, including high penetration, fingerprint spectrum, transience, stability, low photon energy, strong absorption by highly polar molecules, and high bandwidth.^[4,5]^ THz can permeate numerous dielectric materials and nonpolar substances, endowing them with enhanced sensitivity and superior contrast advantages that effectively complement other imaging techniques.^[6]^ Furthermore, THz radiation possesses low photon energy and, as a result, does not induce damage when traversing living tissues, rendering it valuable in the medical domain. Fingerprint spectroscopy arises from the wealth of physical and chemical information encompassed within the THz waveband, facilitating the identification of an object’s composition based on these distinct spectral characteristics. Owing to their exceptional spectral attributes, high spatial resolution, and capacity to penetrate nonpolar molecules, THz technology holds immense potential in the field of disease diagnosis.^[7]^

Since the 1980s, the development of materials and optoelectronic technologies has enabled scholars to harness THz characteristics in the creation of technologies extensively applied in the medical field. These technologies encompass THz biomolecule detection, THz bioimaging detection, THz organism detection, and THz cellular tissue detection.^[8–10]^ In the field of Chinese medicine, several research teams^[11]^ have employed THz technology for the identification of Chinese medicine, investigating acupuncture points, and exploring the essence of Qi in Chinese medicine. These endeavors have yielded significant outcomes, laying the groundwork for further comprehensive research regarding THz applications within the field of Chinese medicine.

## 3. Exploring the inner meaning of Qi theory based on THz technology

The study of “Qi” in Chinese medicine encompasses the conceptualization, production, distribution, and function of “Qi” within the human body, along with its interactions with blood and bodily fluids in the internal organs.^[12–15]^ In the ancient worldview, Qi is defined as a subtle and imperceptible natural substance, considered the source of all phenomena in the universe, and its presence is often demonstrated through motion. Qi is functional, informative, and material and plays propelling, regulating, warming, cooling, defending, consolidating, and mediating roles. In modern times, advanced technological knowledge such as infrared thermography, electromagnetic radiation, and quantum theory has actively been used to understand the nature of Qi. Substantial progress has been achieved, including the Acknowledgments of Qi as a form of energy and elucidating the association of Qi with electromagnetic fields, quantum fields, and dark matter.^[16,17]^ In recent years, some scholars have delved into the exploration and explanation of the THz characteristics of Qi. The findings confirmed the driving and regulatory effects of Qi on the body, corroborated the influence of consciousness on Qi, and further explored its distribution across the body surface.

### 3.1. Qi in the human body has THz characteristics

Ren et al^[18]^ utilized THz detection technology to investigate the THz spectral characteristics of senior Qigong masters during static gong emission, confirming the presence of THz characteristics in the human body’s Qi. THz spectroscopy enables the detection of Qi distribution on the body surface. THz spectroscopy enables the detection of Qi distribution on the body surface. In a study by Zhang et al,^[19]^ this THz detection technique was used to assess numerous acupuncture and non-acupuncture sites on the hands of 50 subjects. Their findings revealed distinct variations in THz energy at acupuncture points compared to non-acupuncture points, implying that Qi is mainly instilled at acupoints on the surface of the human body.

### 3.2. THz detection can offer a theoretical basis for Qi promotion and regulation

In research conducted by Meijun,^[20]^ THz was employed to simulate Qi within the human body and in irradiated human and murine umbilical cord mesenchymal stem cells. The findings demonstrated that low-intensity THz could elevate the positive expression rate of mesenchymal stem cells, significantly increase white and red blood cell numbers, hemoglobin levels, and platelet abundance. It was concluded that THz has a Qi-like pushing effect, which could “stimulate and promote” cell viability, cell proliferation, and hematopoietic function in mice. Du Jing^[21]^ conducted in vitro using human umbilical cord blood mesenchymal stem cells, stimulating human Qi through rubbing hands and observing the effects on the cells. The results showed an increased mesenchymal stem cell proliferation rate, providing evidence that Qi can enhance vitality. Pengfei et al^[22]^ and Na et al^[23]^ recorded THz spectral characteristics at corresponding meridian points in college students before and after Yijinjing and Liuzi Jue exercises. The identification of these meridian points relied on data from infrared and pulse wave detection. Their findings indicated that the practice of Qigong exerts a positive influence on the THz characteristics, providing evidence that this exercise has a regulatory effect on the viscera and meridians of the human body, further refining the concept of Qi regulation.

### 3.3. THz detection can provide a scientific basis for the influence of consciousness on Qi

Nnanan et al^[24]^ assessed the energy metabolism and THz radiation of the meridian Qi apparatus in sad and depressed mice. The results showed that negative emotions such as pessimism and depression could diminish energy metabolism, weaken the THz magnetic wave radiation of the meridian acupoint Qi apparatus, and lead to Qi deficiency syndromes, including decreased physical endurance. This finding substantiates the TCM theory that links “sadness” to the depletion of Qi. Rao Bin^[25]^ conducted research involving 64 college students, measuring THz intensity at the Laogong point on their right hand during Qigong, supplemented by electroencephalogram measurements. The study found an increase in THz intensity at the Laogong point during Qigong practice. Additionally, there was an increase in α brainwave activity and a decrease in θ brainwave activity, signifying enhanced cognitive abilities, information processing, and memory consolidation within the holistic state of form, Qi, and spirit. This aligns with Qigong theories in TCM, particularly the concepts of “guiding Qi by meaning” and “meaning to Qi.” Simultaneously, Zhang et al^[19]^ examined the THz energy intensity at the Hegu point on the right hands of 68 subjects before and after focusing their consciousness. The findings demonstrated distinct variations in THz energy levels after concentrating consciousness compared to baseline measurements before the addition of deliberate thoughts. This further underscores the influence of thoughts on Qi.

## 4. Discussion concerning the connotation of acupoint meridian theory based on THz technology

Meridian theory holds a significant position within traditional Chinese medicine and serves as a foundational theoretical framework. “Meridians are complex physiological networks in the human body, and acupuncture points are the nodes at the intersection of these networks.” In the Yellow Emperor’s Classic of Internal Medicine, meridians are morphologically described as “internal organs and external branches,” and “ move the Qi and blood,” “regulate yin and yang,” and “regulate deficiency.” The functional descriptions of meridians as “moving Qi and blood,” “regulating yin and yang,” “regulating deficiency,” and “dealing with all diseases” reflect the importance of meridians in regulating body balance.^[26]^ Existing research regarding the essence of meridians shows multidisciplinary development involving neurology, biology, physics, and other aspects.^[27]^ The application of THz in the context of meridians and acupoints primarily revolves around its potential to precisely quantify the physiopathological changes within meridians through modern technical means. It serves as a theoretical foundation for understanding the fascial gap as a conduit for meridian Qi. Additionally, it also offers a biophysical basis for substantiating the existence of the meridian Qi, supplying diagnostic parameters for assessing meridian balance, and validating the scientific soundness of disease treatment through the modern scientific understanding of meridians and their influence on the movement of Qi and blood.

### 4.1. Human acupuncture points have THz radiation, and the amount of THz radiation varies from point to point

Huang et al^[28]^ found that each acupoint on the hand has the same biophysical basis but that there are differences in the biophysical properties at the 3 sites. The average radiation spectrum of acupoints on the palm of the hand is greater than that on the back of the hand, and that on the back of the hand is higher than that on both sides of the palm at the red and white flesh interstices. The fascial gap is a meridian Qi channel that provides a scientific basis for this theory. Huang et al^[29]^ found that radiation levels at the Laogong acupuncture point were higher than those at the Shaofu and Outer Laogong points, and that the radiation levels at the Shaofu and Outer Laogong points were closer. At the outer Laogong point, the male acupuncture point had higher radiation levels than the female acupuncture point. The team^[30]^ further found that the THz radiation intensity of the original hand acupuncture points was higher than that of the foot acupuncture points. They proposed that, to some extent, the range of medical reference values of THz radiation intensity of the original acupuncture points could represent an equilibrium state of their corresponding meridians.

### 4.2. THz spectrum characteristics of original acupoints as an index to judge the balance of meridians

Zhang et al^[31]^ verified the feasibility of using the THz characteristics of the original 12 meridians as an index to judge the balance of meridians from theoretical and experimental perspectives. The original point had the characteristics of holography, superior effects, and bidirectional regulation. As an important part of the meridians, it can reflect the physiological and pathological changes of the meridians in a “subtle manner.” The detection and evaluation of the THz wave signal at the original point is an effective reference for determining whether the body is in the balance of the meridians. The team further tested the THz spectral characteristics of the original points of the 12 meridians of the human body. Taking 60 healthy college students as research subjects, the team drew the curve of the THz radiation medical reference values range of the original points of the 12 meridians of the human body.^[32]^ At the same time, it was concluded that the THz radiation of the original acupoint is related to anatomical location, and the THz radiation intensity of the original acupoint of the hand is higher than that of the foot. It was also identified that 3.01 THz may be the frequency band most closely related to human acupoints, the Qi of the human body, and the THz wave. If the detected THz radiation curve frequency exceeds the upper and lower limits of medical reference value ranges, it indicates that some of the measured THz radiation energies of the original acupoints are abnormal and the meridians are in an unbalanced state. This conclusion provides a theoretical basis and data reference for future research and development of THz wave correlation meridian balance instruments.

## 5. Exploring connotations of moxibustion theory based on THz technology

Moxibustion is a treatment method that utilizes moxa strips and pillars made from moxa leaves, which are ignited to target acupuncture points or specific areas of the body. This stimulation adjusts disrupted physiological and biochemical functions within the human body by enhancing the activity of meridian Qi. The ultimate goal is disease prevention and treatment. “Acupuncture and Moxibustion Exploration Fugue” states that “moxibustion refers to the burning of fire on the acupuncture points.” Burning moxa strips apply thermal effects to meridian acupuncture points, aiding in the circulation of Yuan Yang energy and the opening of the meridians.^[33]^

The majority of existing studies have delved into various aspects, including medicinal, chemical, thermal, light radiation, and meridian effects.^[34]^ Furthermore, scholars have explored it from a THz perspective. Liang et al^[35]^ conducted a direct demonstration showing that THz images of the burning areas’ contours could be generated by detecting differences in the moxa stick before and after its water injection. This is accomplished by positioning a polyethylene plastic bag in front of the imaging lens, thus generating THz bands. The existing research also uncovers that moxa burning spectra exhibit THz bands. Factors such as moxa stick diameter, age, material, various moxibustion methods, acupuncture points, moxa stick mass, and maximum burning temperature are correlated with the spectral profile and intensity of THz radiation. Research has confirmed that the essential characteristics of moxa stick material and moxibustion techniques, which influence therapeutic outcomes, may manifest as differences in the generated THz spectrum. Consequently, a THz moxibustion instrument, utilizing the THz spectrum produced during moxibustion, has been developed for clinical use.

Regarding moxa strip material and generated THz, Liang et al^[35]^ further applied the THz technique to compare differences in THz spectra of 4 types of moxibustion involving different diameters, years, and materials and found that the relative intensity of THz after burning moxa strips made of 18 mm diameter moxa, 5-year moxa velvet, and pure moxa velvet was higher than that of moxa strips made of 5 mm diameter moxa, 1-year moxa velvet, and common moxa velvet. Thus, it was concluded that the relative intensity of the THz spectrum produced by burning moxa strips is closely related to the diameter, year, and moxibustion material of the moxa strips, which also provides a theoretical basis for the influence of moxa strip material on the therapeutic efficacy of moxibustion. Deng et al^[36]^ employed thermocouples to measure the maximum temperature achieved over time with various brands of moxa strips. Their research revealed that the greater the THz intensity of different moxa strip brands, the higher the attainable burning temperature. As a result, the duration of moxa strip combustion and the time required to reach maximum temperature could be analyzed based on variations in THz intensity. This leads to the conclusion that THz strength is correlated with both the highest attainable burning temperature and the quality of the moxa column.

In terms of moxibustion techniques and generated THz waves, Zhang Zhounan et al^[37]^ employed THz techniques to compare the THz spectral characteristics of acupuncture points following inter-ginger moxibustion, mild moxibustion, and bird-peck moxibustion. The study included 50 university students and focused on selected acupuncture points, including Yuji, Laogong, Shaofu, Houxi, Zhongzhu, and Hegu points. The results indicated variations in the THz waveforms radiated by different moxibustion techniques. Notably, the bird-pecking technique exhibited a pulse-like THz profile spanning the 0.90 to 2.00 THz band, while the THz spectrum of isolated ginger moxibustion resembled that of human acupoints. Consequently, the study concluded that the THz spectral radiation profile generated by moxibustion is influenced by the specific moxibustion method employed.

In terms of moxibustion affecting homo- and hetero-meridian acupuncture points, Huang Zhijun et al^[38]^ utilized the THz technique to monitor changes in the THz spectral characteristics of ipsilateral Laogong and Shaofu before and after moxibustion of right Neiguan combustion. Their findings revealed that the ipsilateral Laogong and Shaofu THz radiation were lower after right Neiguan compared to before the treatment. Moreover, the difference in THz radiation reduction in ipsilateral Shaofo points before and after right Neiguan moxibustion was more significant than the difference observed in ipsilateral Laogong points. This implies that moxibustion can influence the levels of THz radiation not only at the same meridian points but also at other meridian points.

Moxibustion, consciousness, and internal syndrome observation can enhance THz radiation of acupoints. Huang Zhijun et al^[39,40]^ explored the correlation between zang fu organs and meridians from the THz spectrum perspective. Before and after moxibustion on the 5 zang organs and mu points, 50 healthy college students were tested for THz radiation on the original 5 zang organs and found that 3.584 to 1.290 THz frequencies were the characteristic frequency bands of the original 5 zang organs and meridians. The THz wave radiation intensity of the original 5 zang organs and collaterals was also enhanced after moxibustion, with statistically significant differences. Moxibustion could stimulate the collection points of the zang fu organs and stimulate the sense transmission of the zang fu organs and meridians, thus changing the original meridian points and meridians and providing a biophysical basis for the correlation between the 5 zang organs and the meridian Qi. Zhang et al^[39]^ verified through experiments that consciousness can affect the Qi of the human body and different levels of consciousness can have different effects on the Qi of the human body, thus proving that consciousness can affect the physiological and physical properties of acupoints. Jiancheng et al^[40]^ found that TCM internal syndrome observations can strengthen the smoothness of human meridians, improve the measurement of Qi in different meridians and acupoints of the hand, and thus increase the THz wave radiation of meridians and acupoints. In summary, the discovery that moxibustion, consciousness, and internal syndrome observations can enhance the THz radiation of acupoints not only provides a biophysical basis for the existence of the Qi of meridians but also provides a reference for the research and development of related THz therapeutic instruments.

In clinical practice, Feng et al^[41]^ have designed a THz moxibustion device that replicates the infrared and THz spectra emitted during traditional moxibustion. This device effectively improves the sleep quality of clinical insomnia patients when applied to their Bagua points. By emulating the infrared and THz spectra produced during traditional moxibustion, the THz-moxibustion device operates in a manner consistent with traditional moxibustion in terms of temperature, infrared, and THz waves. The research conducted in the realm of THz technology pertaining to moxibustion, the nature of moxa sticks, and moxa points introduces novel research methodologies and approaches for treatment, detection, and other investigative methods, thereby introducing innovative clinical treatment approaches.

## 6. Conclusions and prospects

THz technology has yielded a series of research achievements in the field of TCM, with a primary emphasis on exploring the interpretations of Qi theory, meridians, acupoints, and moxibustion. THz waves serve as a material foundation to validate and regulate Qi within the human body, offering a biophysical basis for the existence of meridian Qi. They enable the assessment of meridian balance through THz spectrum signals generated by meridians and acupoints. THz technology has confirmed that burning moxibustion sticks emits THz radiation, and the materials of the moxa stick and the moxibustion technique used influence the THz radiation generated. Furthermore, moxibustion^[41]^ and acupuncture,^[3]^ guided by THz spectrum principles, have been applied in clinical practice with successful outcomes.

In comparison to the advancements achieved through THz technology in medical diagnosis, imaging, and biological effects, the research and application of THz technology in TCM are still in the exploratory stage.^[2,11,42]^ Currently, scholars have proposed innovative research avenues for investigating the essence of Qi, meridians, and moxibustion in TCM through the lens of THz technology. This exploration goes beyond Qi theory to encompass the examinations of meridians and acupoints. It has provided evidence of the theory’s scientific validity and feasibility, leading to the development of corresponding THz therapeutic devices for clinical applications. A discourse on theoretical connotations can be integrated with THz spectroscopy, detection, and imaging technology to delve deeper into dynamic changes and uncover the evolving patterns and visceral correlation from a physical standpoint. Currently, many studies focus on the treatment of insomnia in relation to THZ technology. Nevertheless, there remains a need to broaden the scope of diseases under consideration and recognize the diagnostic and therapeutic advantages offered by THz.

## Acknowledgments

The authors thank Editage for polishing the language of the manuscript.

## Author contributions

**Conceptualization:** Wen-chun Zhang, Guang-rui Huang, Shao-hui Geng.

**Funding acquisition:** Guang-rui Huang.

**Investigation:** Li Liu.

**Supervision:** Wen-chun Zhang.

**Validation:** Jian-cheng Liu, Wen-chun Zhang.

**Visualization:** Zhi-min Lin, Xin Liu.

**Writing – original draft:** Shao-hui Geng, Li Liu, Zhi-min Lin, Hui Zhang, Ri-geng Mei, Jian-cheng Liu.

**Writing – review & editing:** Shao-hui Geng, Jian-cheng Liu, Guang-rui Huang, Wen-chun Zhang.
